# Interrelationship of Seasons with Inflammation, Red Meat, Fruit, and Vegetable Intakes, Cardio-Metabolic Health, and Smoking Status among Breast Cancer Survivors

**DOI:** 10.3390/jcm10040636

**Published:** 2021-02-07

**Authors:** Tianying Wu, Rajashree Shinde, Robert Castro, John P. Pierce

**Affiliations:** 1 Division of Epidemiology and Biostatistics, School of Public Health, San Diego State University, San Diego, CA 92182, USA; rajashreeshinde36@gmail.com; 2 Moores Cancer Center, School of Medicine, University of California, San Diego, CA 92093, USA; jppierce@health.ucsd.edu; 3 School of Exercise and Nutritional Sciences, San Diego State University, San Diego, CA 92182, USA; robertjamescastro@hotmail.com

**Keywords:** season, diet, inflammation, time to eat, meat, vegetable and fruit, past smokers, breast cancer survivors

## Abstract

Seasons can affect human inflammatory status and the occurrence of diseases, and foods may also have differential impacts on inflammation across seasons; however, few studies have investigated whether there are independent and joint impacts of seasons and red meat, fruit and vegetable intakes on inflammation in breast cancer survivors. We conducted a cross-sectional study by leveraging a large cohort, the Women’s Healthy Eating and Living (WHEL) study. The WHEL study comprised primarily early stage breast cancer survivors and collected blood samples, dietary intake, demographic, and health status information at baseline. We selected 2919 participants who provided baseline dietary information and had measurement of C-reactive protein (CRP), a general marker of inflammation. In our multivariable-adjusted analyses, we found that red meat intakes were positively associated, while fruit and vegetable intakes were inversely associated with CRP; blood collected in the winter season was associated with lower CRP when compared to summer; and increased smoking intensity and body mass index (BMI) as well as having cardio-metabolic conditions (such as heart disease or diabetes) were positively associated with CRP. Furthermore, we examined the joint associations of food intakes and the season of blood draw with CRP in different subgroups. We found that moderate intakes of red meat were associated with a reduction of CRP in winter but not in other seasons; increased intakes of fruit and vegetables were associated with reduced inflammation in most seasons except winter. These associations were observed in most subgroups except past smokers with pack-years ≥ 15, in whom we observed no benefit of red meat intakes in winter. Our study provides valuable evidence for considering seasonal impacts on inflammation and seasonal food impacts in different subgroups among breast cancer survivors. The results of our study are in line with one of the emphases of the current NIH 2020–2030 nutrition strategy plan—namely, pay attention to what, when, and who should eat.

## 1. Introduction

Seasons have impacts on human physiology [1,2] and such seasonal impacts may vary by individuals [3]; endogenous health conditions can be an important determining factor for seasonal impacts. For instance, inflammation, as measured by C-reactive protein (CRP), was higher in winter than in summer in healthy people, people with osteoarthritis, and hemodialysis patients [4,5,6,7]; however, among people with rheumatoid arthritis and stroke, the trend was either the opposite [7] or showed no difference between seasons [8].

The seasonal impacts on human physiology can also lead to various consumptions of food intakes, and dissimilar responses to the same food also differ across seasons. For instance, metabolic rate is generally higher in winter than in summer [3], thereby leading to a higher demand for energy intakes in winter. Hence, foods that can produce more energy and heat will help people acclimatize to winter. Red meat is a thermogenesis food [9]; it is enriched with energy and protein, which are critical for thermogenesis [10]. Total red meat consumption was found to be highest in winter when compared to other seasons [11,12]; on the other hand, fruit and vegetable intakes were found to be higher in summer than during the rest of the year [13]. Understanding whether the impacts of food on aging-related biomarkers (e.g., inflammation) are different across seasons and whether these impacts vary by people with different metabolic conditions will help us design an optimal diet to prevent aging.

To date, no studies have examined the seasonal impacts on inflammation in breast cancer survivors. Breast cancer survivors experience accelerated aging due to the cancer itself or the cancer treatment [14,15]; thus, it is important to study this population. Furthermore, whether seasonal impacts on inflammation are distinct across survivors with different cardio-metabolic conditions, past smoking intensities, and whether the impact of red meat and fruit and vegetable intakes on inflammation varies by seasons and by diverse subgroups, have never been studied. As we know, past smokers account for more than 35–40% of breast cancer survivors [16,17], and women with cardio-metabolic conditions account for more than 30% [18,19] of breast cancer survivors; thus, these people experience a higher rate of aging and are more susceptible to environmental challenges than breast cancer survivors without these conditions. Understanding the seasonal impacts on breast cancer survivors as a whole and in various subgroups will provide better prevention strategies. This study sought to examine the seasonal impacts on CRP in breast cancer survivors to determine whether the pattern of seasonal change for CRP would vary by past smoking intensity, cardio-metabolic health condition, obesity status, and intakes of red meat, vegetables, and fruits.

## 2. Materials and Methods

### 2.1. Study Design

We leveraged a large cohort of breast cancer survivors, the Women’s Healthy Eating and Living (WHEL) study, to conduct the current study. Between 1995 and 2000, 3088 early-stage (stage I, II, or IIIA) breast cancer survivors within 4 years of diagnosis were enrolled in the WHEL study. These women were recruited from multiple clinical centers located in California, Arizona, Texas, and Oregon. The WHEL study was initially a randomized trial designed to test whether a diet low in fat and rich in vegetables, fruit, and fiber improved breast cancer prognosis. Extensive details regarding inclusion and exclusion criteria can be found in previous publications [20]. Briefly, inclusion criteria were as follows: women who had stage I (≥1 cm), II, or IIIA breast cancer diagnosed within the previous 4 years, were 18–70 years old at diagnosis, had completed primary therapy, had no evidence of cancer recurrence, did not have life-threatening comorbidities, and were able to provide information about their dietary intakes via 24 h food recall. We excluded women who were insulin-dependent, required a special diet, were diagnosed after age 70, and had stage 1 tumors smaller than 1 cm.

For this study, we only used baseline data, which were collected before intervention. Therefore, our study was a cross-sectional study and the information related to intervention will not matter. The Institutional Review Board (IRB) at the University of California at San Diego and other participating medical centers approved the original study. All subjects provided written informed consent. We used the de-identified data provided by the principal investigator of the WHEL study, thus the exempt IRB was approved by the San Diego State University IRB committee (protocol number: Temp-1286).

### 2.2. Dietary Assessment

At baseline, dietary intakes were assessed by 24 h dietary recalls. There were four prescheduled, 24 h dietary recalls collected by telephone on random days over a 3 week period: two on the weekends and two during weekdays. We used the multi-pass software-driven recall protocol of the Nutritional Data System software (NDS-R, 1994–2006, 91 University of Minnesota, Minneapolis, MN, USA) to analyze the foods and nutrients.

Of note, recalls were not conducted at the same time across all women. For instance, some women may finish their recalls in summer and others finish them in winter or other seasons. Within one person, the four 24 h recalls were generally finished in one month.

### 2.3. Season of Blood Draw at Baseline and Measurement of CRP Using Baseline Blood Samples

At baseline, blood samples were collected from all participants across four seasons in 1995–1996. Approximately 70% of women provided blood samples and completed 24 h recalls during the same season (see Supplemental Table S2). The serum concentration of CRP was measured via a high-sensitivity electrochemiluminescence assay (MesoScale Discovery, Gaithersburg, MD, USA) by the clinical biochemistry lab at the University of Vermont. The inter-assay coefficients of variation ranged between 7% and 12%. The lower detection limit was 0.02 mg/L.

### 2.4. Smoking Assessment

A brief smoking history questionnaire was administered to participants at baseline. The questionnaire included age of smoking initiation and cessation, duration of smoking, and the number of cigarettes/day. We classified a lifetime history of <100 cigarettes as never smoking. Women who reported having quit smoking at the baseline survey were classified as past smokers; women who still smoked at the baseline survey were classified as current smokers. All ever smokers reported their intensity of smoking (cigarettes/day) and the number of years they smoked regularly. Pack-years exposure was determined by multiplying duration of smoking by intensity. One pack-year is equal to smoking one pack per day for one year or two packs per day for half a year.

### 2.5. Other Assessments

Demographic characteristics and health status, including a series of comorbid conditions (e.g., diabetes, cardiovascular diseases, osteoporosis, and medications such as diabetic and cardiovascular medications), were self-reported. Cancer diagnosis and treatment information were extracted from medical records (e.g., tumor stage, size, hormone receptor status, and use of radiation, chemotherapy, and/or post-treatment anti-estrogens use). We used a questionnaire validated in the Women’s Health Initiative to assess physical activity levels [21], which were calculated by metabolic equivalent tasks (METs), as previous studies did [22]. For our analyses, we classified women with and without cardio-metabolic conditions. We defined individuals with cardio-metabolic conditions as anyone who had been told by their doctors that they had cardiovascular, diabetic, or insulin resistance-related conditions or who used medications to lower blood cholesterol or blood pressure or treat cardiovascular and diabetic conditions.

### 2.6. Statistical Analyses

The data analysis was completed using SAS 9.4 (SAS Institute Inc., Cary, NC, USA). We used linear regression models to assess the associations between independent variables (e.g., food intakes and season) and the dependent variable (i.e., CRP). CRP was log-transformed because it was not normally distributed. Of note, in addition to food intakes (red meat, vegetable, and fruit intakes), season of blood draw, smoking status, and cardio-metabolic condition were also treated as main exposure variables in Table 2.

Food intakes (red meat, vegetable, and fruit intakes) were categorized into quartiles and treated as independent variables. In addition to the main exposure variables, we adjusted for the following covariates in the multivariable models based on a priori assumption: age at enrollment, ethnicity, physical activities, body mass index (BMI), total caloric intake, processed meat intakes, menopausal status, and cancer characteristics—namely, tumor stage (I, II, IIIA), hormonal receptor status, use of tamoxifen, radiation, and chemotherapy.

We further evaluated the joint impacts of diet and season of blood draw on CRP. Finally, we assessed the joint impacts of a summary score of selected foods and season of blood draw on CRP in different subgroups (e.g., women with and without cardio-metabolic condition). Our food summary score was created based on three types of food: fresh red meat intake, vegetable intake, and fruit intake based on their associations with CRP. Women with red meat intake > quartile 1 were assigned “1” and at quartile 1 assigned “0”; women with fruit intake ≥ quartile 4 were assigned “−1” and < quartile 4 were assigned “0”; and women with vegetable intake ≥ quartile 4 were assigned “−1” and < quartile 4 were “0.” The summary score ranged from −2 to 1. The cut-points for assigned score were based on the dose–response associations observed in Table 2.

In order to have sufficient power, for our main analyses, we examined the associations of food intakes with serum CRP regardless of when the 24 h recalls were collected (results in Tables 2–4); hence, only the season of the blood draw was considered in those analyses. We limited our sensitivity analyses to those women who provided 24 h recalls and blood samples during the same seasons; hence, the season of food intakes and the season of blood draw were both considered. We compared the results in the sensitivity analyses to our main analyses to determine whether they were consistent.

## 3. Results

### 3.1. Baseline Characteristics by Disease Outcomes

As shown in Table 1, the majority of survivors were white and postmenopausal women, had chemotherapy and radiation therapy, and used tamoxifen. Approximately 95% had stage I or II tumors, 62% were estrogen receptor- and progesterone receptor-positive (ER+/PR+); there were 53% never smokers, 40% past smokers, and 43% normal weight women. More than half of the women lived in Northern California or Oregon, approximately 17% lived in Southern California and 27% lived in Arizona or Texas. The times of blood draws were almost evenly distributed across the four seasons, with slightly higher percentages in summer and fall. The median age at enrollment was 52. The median intake of unprocessed red meat was 72 g/day, of vegetables was 2.57 servings/day, and of fruits was 2.07 servings/day. In addition, we provided selected characteristic variables based on seasons and red meat, fruit, and vegetable intakes plus another 28 food components according to seasons in Supplemental Table S1. As this table indicates, physical activity and several food and nutrient intakes are significantly or marginally different across seasons, including total calories, vegetable and fruit intakes, and intakes of refined grain, zinc, riboflavin, niacin, folate acid, potassium, magnesium, iron, vitamins A and C, and alpha-tocopherol (*p* ≤ 0.07).

### 3.2. Seasons for Which Dietary 24 h Recalls and Blood Draw Were Performed

As shown in Supplemental Table S2, the collection of dietary 24 h recalls was almost evenly distributed across four seasons (slightly more in summer). Women who had blood draws and 24 h recalls collected during the same seasons accounted for approximately 70% (the sum of column 3 in Supplemental Table S2) of the total population.

### 3.3. Associations of Food Intakes, Season of Blood Draw, and Other Characteristics with Serum CRP in the Whole Data Set

As shown in Table 2, fresh red meat was positively associated and vegetables and fruits were inversely associated with serum CRP (*p* for trend <0.001 for all of these associations). Furthermore, the positive associations between red meat and serum CRP seemed to plateau after quartile 1, whereas the inverse associations between vegetable and fruit intakes and CRP did not reach statistical significance until at or above quartile 4. With regard to season of blood draw, CRP was 11% lower (beta = −0.11; *p* = 0.04) if the blood samples were collected in winter than in summer. Increased BMI was significantly associated with increased CRP; women with BMI ≥ 30 had 171% higher CRP than normal weight women (beta = 1.71; *p* < 0.0001). Women with cardio-metabolic conditions had higher CRP than women without cardio-metabolic conditions (beta = 0.12; *p* = 0.01). Meanwhile, past smokers with a higher smoking intensity (pack-years ≥ 15) had higher CRP than never smokers (beta = 0.14; *p* = 0.02).

The cut-points for classifying categories of red meat, vegetable, and fruit intakes in Table 3 were based on the dose-response associations observed in Table 2. For instance, we did not observe a further increase of the association between red meat and CRP after quartile 1; thus, we used quartile 1 as the cut-point. Cut-points for other foods were set the same way as red meat. In Table 3, compared to women with higher red meat intakes (higher than quartile 1) who had blood draws in summer, women with lower red meat intakes (lower than at quartile 1) who had blood draws in the fall, winter, or spring had a 21% to 22% reduction of CRP (*p* < 0.05 for all these associations). Interestingly, among women with higher red meat intakes, when blood was collected in winter, CRP was 11% lower (*p* = 0.10) than when collected in summer. In terms of fruit intakes, women with higher fruit intakes (at quartile 4) who had a blood draw in any of the four seasons had 18% to 21% lower CRP (*p* ≤ 0.05 for summer, fall, and winter; *p* = 0.11 for spring) compared to women with lower fruit intakes (lower than quartile 4) who had a blood draw in summer. Interestingly, among women with lower fruit intakes, CRP was 15% lower (*p* = 0.02) if blood was collected in winter rather than summer. Regarding vegetable intakes, compared to women with lower vegetable intakes (lower than quartile 4) who had a blood draw in the summer, women with higher vegetable intakes (at quartile 4) who had a blood draw in the summer or spring showed a 15% to 16% reduction of CRP (*p* = 0.06 for summer; *p* = 0.11 for spring). Interestingly, and similar to fruit intake results, among women with lower vegetable intakes, CRP showed a 17% reduction (*p* = 0.007) if blood was collected in winter rather than summer.

### 3.4. Joint Associations of Food Summary Score and Season of Blood Draw with Serum CRP in Different Subgroups

There are multiple subgroups, two categories of food summary scores, four seasons of blood collection, and six groups of women with different smoking, obesity, or cardiovascular conditions (Table 4). A higher food summary score has higher inflammatory potential. Using women with a higher food score who had a blood collection in the summer as the reference group, we did two types of comparisons, as detailed next. The results shown in Table 4 highlighted an important message: Past smokers with higher smoking intensity (pack-years ≥ 15) have different patterns compared to the remaining subgroups. Furthermore, current smokers were excluded from the analyses conducted for Table 4.

#### 3.4.1. Comparison of CRP Levels between Blood Draws in Non-Summer Seasons and Those in Summer, among Women with a Higher Food Inflammatory Score (Score of 0–1)

In most subgroups, CRP was associated with a 14% to 21% reduction (*p*-values ranged from 0.03 to 0.09) if blood was collected in winter compared to summer. There were two exceptions: Among obese women, CRP decreased if blood was collected in the fall compared to summer, and among past smokers with pack-years of smoking ≥15, no significant difference was found when comparing blood collected in other seasons to that in summer.

#### 3.4.2. Comparison of Women with a Lower Food Inflammatory Score (Score of −1 to −2) Who Had Blood Draws in Different Seasons to Women with a Higher Food Inflammatory Score (Score of 0 to 1) Who Had a Blood Draw in Summer

Compared to women with a higher food inflammatory score who had a blood draw in summer, a low food inflammatory score was associated with a 20% to 50% decrease in CRP (*p*-values ranged from 0.001 to 0.06) if blood was collected in spring and/or summer in most subgroups, except for past smokers with pack-years of smoking ≥15 as CRP in this group was reduced when blood was collected in the fall and winter (*p* ≤ 0.08).

### 3.5. Joint Associations of Food Inflammatory Score and Season of Blood Draw with Serum CRP in the Whole Data Set (Excluding Past Smokers with Pack-Years ≥15)

**Figure 1:**
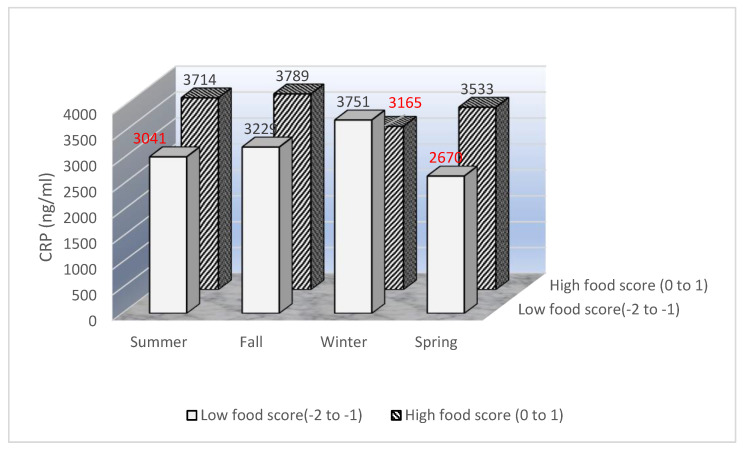
Joint associations of food summary score and season of blood draw with serum C-reactive protein (CRP). Covariates in the linear regression model included age at diagnosis, race/ethnicity, education level, menopausal status at baseline, total calorie intake, physical activity, body mass index, cardio-metabolic condition, smoking status (never smokers and past smokers), tumor stage, estrogen and progesterone receptor status, tamoxifen use, radiation and chemotherapy, and states of residence (Northern California, Southern California, Arizona and Texas). Note: high food score denotes high food inflammatory score; low food score denotes low food inflammatory score; bars with data labeled in red means that *p*-values were significant (<0.05) comparing to the reference bar (i.e., the bar with CRP = 3714 ng/mL).

As most subgroups showed similar trends in Table 4, we combined all the subgroups excluding current smokers and past smokers with pack-years ≥15 and examined the joint associations of food intakes and season of blood draw with serum CRP (Figure 1). As shown in Figure 1, the overall patterns were similar to the main findings in Table 4. Compared to the reference group which is the group with a higher food inflammatory score (score of 0 to 1) and had blood draw in summer, women with a higher food inflammatory score and who had a blood draw in winter had 15% lower CRP (*p* = 0.03) and women with a lower food inflammatory score (score of −2 to −1) and had blood draws in summer and spring had 18% lower (*p* = 0.04) and 28% lower CRP (*p* = 0.003), respectively. To minimize the residual confounding by other pro- and anti-inflammatory food components, we also adjusted for 28 food components that were previously used to create the dietary inflammatory index [23,24]. The 28 food components are: refined grain, whole grain, glucose, dietary fiber, total saturated fat, total monounsaturated fat, trans fatty acids, n6/n3 fatty acid ratio, cholesterol, zinc, selenium, thiamin, riboflavin, niacin, folate acid, potassium, magnesium, iron, vitamin A, vitamin C, vitamin B6, vitamin B12, vitamin D, beta carotene, alpha carotene, alpha tocopherol, caffeine and alcohol intake. The results of the overall pattern were maintained, although CRP levels in women who had a lower food inflammatory score and blood draws in summer were no longer statistically different from the reference group. The magnitude of the reduction of CRP was similar for women who had a higher food inflammatory score (score of 0 to 1) and had blood draws in winter (14% reduction; *p* = 0.04) but decreased for the other two groups with a lower food inflammatory score: a 10% reduction (*p* = 0.35) in women with blood draws in summer and a 24% reduction (*p* = 0.04) in women with blood draws in spring.

### 3.6. Sensitivity Analyses Limited to People Who Provided 24 h Recalls and Blood Samples during the Same Seasons

Limiting analyses to people who provided dietary 24 h recalls and blood samples during the same season may help better determine the seasonal food impacts on CRP levels. We examined the joint associations of food intakes and season of blood draw with serum CRP within this group (see Supplemental Table S3). The overall patterns were very similar to results shown in Table 3, although some of the *p*-values were less significant (*p*-values were in the 0.00009 to 0.14 range).

Furthermore, as shown in Supplemental Table S4, we conducted similar analyses to those in Figure 1 among the whole data set, while excluding current smokers and past smokers with pack-years ≥15. The results were similar to the overall associations observed in most subgroups in Table 4, although the *p*-values were less significant.

## 4. Discussion

In general, breast cancer survivors had 11% lower CRP levels in winter than in summer. Higher smoking intensity, a cardio-metabolic condition, and higher BMI were positively associated with increased CRP. Although the fresh red meat intakes were positively associated and fruit and vegetable intakes were inversely associated with CRP in the whole data set, this overall pattern changed with seasons and subgroups. For most subgroups, a moderate increase in red meat intakes may be beneficial (reduction of inflammation) in winter but not in other seasons and increased intakes of fruits and vegetables were associated with reduced inflammation in most seasons except winter. Past smokers with higher smoking intensity (≥15 pack-years of smoking) had different patterns compared to other groups; in past smokers with high past smoking intensities, moderate increases in intakes of fresh red meat were not associated with a reduction in inflammation in any season, but were associated with an increased inflammation in spring; higher intakes of fruit and vegetables were associated with reduced inflammation in winter, which is in contrast with most subgroups.

In this study, we used the reduction of CRP as a benefit index. In winter, we found that breast cancer survivors may benefit more from having a moderately increased intake of red meat than a low intake of red meat; this was observed in most subgroups except past smokers with higher smoking intensity. Red meat intakes higher than 25% in the summer were associated with an 11% reduction of CRP in winter in the whole data set (Table 3). A food inflammatory score of 0–1, which represents a diet with more red meat than fruits and vegetables, was associated with a 14% to 21% reduction of CRP if blood was collected in winter compared to summer in most subgroups (*p*-values were significant or marginally significant; see Table 4), whereas a food inflammatory score of −2 to −1, which represents a diet with more fruits and vegetables than red meat, had no impact on CRP in winter compared to women with a food inflammatory score of 0–1 who had a blood draw in summer. Red meat may offer several unique benefits in winter. Humans’ metabolic rate is 64% higher in winter than in summer [3]. This increased metabolic rate is necessary to generate the heat needed to respond to the cold environment [3]. Red meat is rich in protein, which induces higher thermogenesis than carbohydrates and fat [9]. Thermogenesis is not just for acclimating to cooler weather, but also for maintaining normal physical functions. For instance, blood pressure is elevated in winter and blood vessels are stiffer in cold conditions. Appropriate thermogenesis is important for normal blood circulation [25,26]. Furthermore, animal protein (e.g., pork) promotes more energy expenditure than plant-based protein (e.g., soy) [27], promotes muscle anabolism, and favors the retention of lean muscle mass [9,10,28,29], which can lead to weight loss and improve insulin-sensitivity mass [29,30]. As such, a moderate intake of red meat in winter does have several advantages, and its function cannot be replaced by intakes of fruits and vegetables. Improving metabolic health, which is influenced by thermogenesis, insulin sensitivity, normal physiological function, and energy expenditures, will certainly reduce inflammation [31,32].

In contrast to other subgroups, among past smokers with higher intensity (>15 pack-years), a high food inflammatory score was not associated with a reduction of CRP in winter. Although the mechanism has not been well studied, we propose the following explanations. Red meat is a high acid-producing diet, and we found that acid-producing diets were associated with a higher CRP in past smokers than in never smokers [33]. Past smokers have a reduced capacity to excrete excess acid due to their impaired lung and renal function [33,34,35]. Furthermore, past smokers with a higher smoking intensity will have a lower capacity to excrete excess acids than past smokers with a lower smoking intensity. Hence, the benefits of red meat will also depend on a person’s capacity to excrete acids.

In this study, we found that increased fruit and vegetable intakes were associated with a reduction of inflammation in summer and/or spring but not in winter for most subgroups. In summer and spring, people tend to have increased outdoor physical activities [36]; increased outdoor physical activities plus higher temperatures lead to increased sweating and breathing [37,38,39], which can result in more water loss [40]. Approximately 1000 mL/day more water loss through sweat occurs in summer than in winter [41]. Water, fruit, and vegetable consumptions significantly increase in summer compared to winter [13,41,42]. Fruits can help keep the body hydrated, maintain electrolyte balance, and avoid dehydration in summer [41]. Dehydration and electrolyte imbalance promote inflammation [43,44]. Thus, fruits and vegetables can reduce inflammation not only through their antioxidant and polyphenol ingredients [45,46], but also through their restoration of water loss and electrolyte balance function, which may not be well known but is important for humans’ adjustment to seasons.

The American Cancer Society (ACS) and World Cancer Research Fund recommended that breast cancer survivors follow a dietary pattern that is high in vegetables, fruits, whole grains, and legumes; low in saturated fats; and limited in alcohol consumption [47,48,49]. However, these guidelines did not target individualized nutrition. For instance, we previously demonstrated that past smokers with higher past smoking intensity (pack-years ≥ 15) had different susceptibility to an acid-producing diet than never smokers and past smokers with a lower past smoking intensity [50]. Our previous paper [50] and the current paper both suggest that we need to consider the impacts of foods in the context of who will consume the diets; in other words, the impacts of foods will be different among people with different smoking statuses. Furthermore, we focused on red meat instead of other types of meat because it is one of the most important and controversial topics. Although the International Agency for Research on Cancer (IARC) concluded that red meat is a Group 2A carcinogen, which means “probably carcinogenic to humans,” results from observational studies on red meat and cancer were not consistent for most cancers except for colorectal cancer [51,52,53,54,55,56,57]. The ACS and World Cancer Research Fund websites recommend that people should eat no more than a moderate amount of red meat. This recommendation is correct, yet it may overlook the potential benefits of consuming a small amount of red meat in winter. It may also miss the feature that red meat may have different impacts on people with different smoking statuses [56]. Moreover, as previously discussed, red meat may improve thermogenesis and insulin sensitivity and favor the retention of muscle mass [9,10,29,30]; hence, the benefits of red meat in winter may not be limited to inflammation, and more comprehensive studies regarding the differential impacts of red meat on multiple biomarkers among different subgroups across seasons are needed. Nevertheless, people should be aware that red meat is rich in protein, which has acid-producing potential; therefore, consuming even a small amount of red meat should be done together with sufficient fruits and vegetables as minerals such as potassium and magnesium in fruits and vegetables can help balance the acids [58,59,60].

Our study has several strengths. First, this is the first study to assess the benefits of red meat, fruit, and vegetable intakes in different seasons and subgroups in breast cancer survivors. One of the emphases of the NIH 2020–2030 nutrition strategy plan is to study not only what we eat, but also when we eat, as well as individual variabilities. Our study addresses all three features, which can be briefly known as “what, when, and who.” We demonstrated the differential associations of red meat, fruits, and vegetables (“what”) with inflammation in different seasons (“when”) and in different subgroups (“who”). Regarding “when,” existing studies have focused on timing in a day, but not a season during the year. Second, the large sample size (*n* = 2919) allowed us to adjust for multiple covariates and conduct subgroup analyses to determine differential impacts across seasons and subgroups.

Our study also has several limitations. First, our study is a cross-sectional study; thus, causal relationships cannot be demonstrated and the proposed benefits of red meat in winter should be interpreted with caution. Future studies should determine the long-term joint impacts of season and food across subgroups in breast cancer survivors. Second, our study did not include the exact outdoor and indoor temperatures for the blood draw dates; thus, we cannot interpret our results as temperature impacts. Nevertheless, in general, the average temperatures in summer are always higher than those in winter. Third, our main results were based on 24 h recalls taken in different seasons. Some were collected in summer while others were collected in winter or other seasons. Ideally, we should collect food intake information across all four seasons within the same individual; however, this will require collecting sixteen 24 h recalls in the same individual (4 recalls in each season × 4 seasons) among approximately 3000 participants, which may not be realistic. Most cohorts do not even have four 24 h recalls among the same individual. Even so, as shown in Supplemental Table S2, the majority of women (70%) provided their 24 h recalls and blood samples during the same seasons, which allowed us to compare seasonal impact. When we limited our analysis to women who provided recalls and blood samples during the same seasons, joint associations of food summary score and season of blood draw with CRP were similar, as shown in Supplemental Table S4. Fourth, our participants were recruited from different locations, including Northern California, Oregon, Southern California, Arizona, and Texas. These places have different climates as the seasonality is less obvious in Southern California when compared to other places. When we exclude Southern California or limited our analysis to only Arizona or Texas, which have four distinct seasons, the results were more or less similar to the overall results. Finally, our food summary score was created not based on an entire list, but rather on only three food groups (red meat, fruits, and vegetables). Studies on whole dietary patterns in this area are needed in the future. Currently, we measured only CRP in baseline serum samples; more pro-inflammatory (e.g., IL-6) and anti-inflammatory (e.g., IL-10) biomarkers should be measured if we want to comprehensively study the associations of these foods with inflammation and season.

## 5. Conclusions

Our study is the first study to demonstrate seasonal impacts of red meat, fruit, and vegetable intakes on inflammation in different subgroups among breast cancer survivors. In most subgroups, increased intakes of fruits and vegetables are beneficial in most seasons, especially summer and spring. In winter, a moderate intake of red meat (higher than 23 g/day) is more beneficial than no or lower intakes of red meat in most breast cancer survivors except past smokers with a higher smoking intensity (>15 pack-years). According to the 2020–2030 NIH nutrition strategies, we need to pay attention to not only what we eat, but also when we eat and who should eat in order to receive the most benefits from foods. Seasonal impact is an important component to be added to “when we eat”; discrepancies between most groups and past smokers with higher smoking intensity highlight the importance of “who should eat”. Our study identifies three key elements to consider for breast cancer survivors: “what, when, and who”. This should be an important future direction for precision care and individualized nutrition for cancer survivors.

## Figures and Tables

**Table 1:** Baseline characteristics (*n* = 2919).

Characteristics	All Participants
Age at enrollment (years) ^a^	52 (47–59)
Whites (%)	85.7
Body mass index (%)	
Normal weight	42.8
Overweight	31.0
Obese	26.2
Location (%)	
Northern California and Oregon	55.4
Arizona and Texas	27.2
Southern California	17.4
Season of blood draw (%)	
Summer	27.2
Fall	26.8
Winter	22.8
Spring	23.2
Smoking status (%)	
Never smokers	53.4
Past smokers with pack-years <15	26.2
Past smokers with pack-years ≥15	14.6
Current smokers	4.3
Postmenopausal women (%)	79.6
Physical activity (MET/week)	600 (150–1250)
Chemotherapy (%)	69.4
Radiation (%)	61.7
Hormone receptor status (%)	
ER+/PR+	62.4
ER−/PR−	19.9
Cancer stage at diagnosis (%)	
I	39.2
II	56.1
IIIa	4.7
Tamoxifen use (%)	66.5
C-reactive protein (ng/mL)	1759 (673–4240)
Total calorie intakes (kcal)	1685 (1432–1973)
Unprocessed red meat intakes (g/day)	120.83 (66.2–235.9)
Processed meat intakes (g/day)	44.4 (18.9–90.9)
Total vegetable intakes (servings/day)	2.57 (1.7–3.7)
Total fruit intakes (servings/day)	2.07 (1.1–3.3)

^a^ indicates that continuous variables are presented as median (inter-quartile range).

**Table 2:** Food, season of blood draw, and other characteristics in relation to C-reactive protein.

Exposure Variables	Beta (*p*-Value)	Beta (*p*-Value)
	Age-Adjusted	Multivariable-Model
Fresh red meat		
Quartile 1 (0 to <23.1 g/day)	Ref	Ref
Quartile 2 (23.1 to <72.0 g/day)	0.25 (0.0008)	**0.13 (0.05)**
Quartile 3 (72.0 to <174.3 g/day)	0.47 (<0.0001)	**0.16 (0.01)**
Quartile 4 (≥174.3 g/day)	0.66 (<0.0001)	**0.13 (0.05)**
Vegetable intakes		
Quartile 1 (0 to <1.7 servings/day)	Ref	Ref
Quartile 2 (1.7 to <2.6 servings/day)	−0.20 (0.003)	−0.09 (0.12)
Quartile 3 (2.6 to 3.7 servings/day)	−0.31 (<0.0001)	−0.07 (0.16)
Quartile 4 (≥3.7 servings/day)	−0.47 (0.0001)	**−0.12 (0.04)**
Fruit intakes		
Quartile 1 (0 to 1.1 servings/day)	Ref	Ref
Quartile 2 (1.1 to 2.1 serving/day)	−0.10 (0.12)	−0.03 (0.12)
Quartile 3 (2.1 to 3.3 servings/day)	−0.03 (0.16)	−0.03 (0.16)
Quartile 4 (≥3.3 servings/day)	−0.18 (0.003)	**−0.18 (0.003)**
Season of blood draw		
Summer	Ref	Ref
Fall	0.04 (0.58)	0.00 (0.9)
Winter	−0.08 (0.21)	**−0.11 (0.04)**
Spring	−0.03 (0.61)	−0.06 (0.25)
Body mass index		
<25 (kg/m^2^)	Ref	Ref
25–29.9 (kg/m^2^)	0.89(<0.0001)	**0.78 (<0.0001)**
30–34.9 (kg/m^2^)	1.54 (<0.0001)	**1.36 (<0.0001)**
≥35 (kg/m^2^)	1.97 (<0.0001)	**1.71 (<0.0001)**
Having cardio-metabolic condition		
No	Ref	Ref
Yes	0.48 (0.0001)	**0.12 (0.01)**
Smoking status		
Never smoker	Ref	Ref
Past smoker with pack-years 0 to <15	−0.06 (0.09)	−0.08 (0.09)
Past smoker with pack years ≥ 15	0.27 (0.0002)	**0.14 (0.02)**
Current smoker	0.29 (0.01)	0.13 (0.17)

In addition to the variables adjusted in the table, the multivariable-adjusted model also included age, education level, menopausal status at baseline, total calorie intake, processed meat, physical activity, tumor stage, estrogen and progesterone receptor status, tamoxifen use, radiotherapy, chemotherapy, and states of residence (Northern California, Southern California, Arizona and Texas). Note: values in bold indicate that they are statistically different from the reference group in the multivariable-adjusted model. 3.4. Joint Associations of Food Intake and Season of Blood Draw with Serum CRP in Whole Data Set.

**Table 3:** Joint associations of food intakes and seasons with C-reactive protein.

		Beta (*p*-Value)	Beta (*p*-Value)	Beta (*p*-Value)	Beta (*p*-Value)
		Summer	Fall	Winter	Spring
Red meat intakes	>Quartile 1 (≥23 g/day)	Ref	0.06 (0.4)	**−0.11 (0.11)**	−0.03 (0.7)
		*n* = 692	*n* = 656	*n* = 558	*n* = 590
	=Quartile 1 (0 to <23 g/day)	−0.07 (0.5)	**−0.21 (0.04)**	**−0.22 (0.04)**	**−0.21 (0.04)**
		*n* = 103	*n* = 127	*n* = 107	*n* = 85
Fruit intakes	<Quartile 4 (0 to 3.25 servings/day)	Ref	−0.01 (0.86)	**−0.15 (0.02)**	−0.10 (0.14)
		*n* = 541	*n* = 537	*n* = 544	*n* = 559
	=Quartile 4 (3.25 to 11.17 servings/day)	**−0.21 (0.01)**	**−0.20 (0.01)**	**−0.21 (0.05)**	**−0.18 (0.11)**
		*n* = 253	*n* = 245	*n* = 117	*n* = 115
Vegetable intakes	<Quartile 4 (0 to <3.68 servings/day)	Ref	−0.03 (0.69)	**−0.17 (0.007)**	−0.09 (0.15)
		*n* = 583	*n* = 575	*n* = 508	*n* = 515
	=Quartile 4 (3.68 to 16.18 servings/day)	**−0.16 (0.06)**	−0.10 (0.25)	−0.09 (0.37)	**−0.15 (0.11)**
		*n* = 211	*n* = 207	*n* = 153	*n* = 159

Covariates in the linear regression model included age at diagnosis, race/ethnicity, education level, menopausal status at baseline, total calorie intake, physical activity, body mass index, cardio-metabolic condition, smoking status (never smokers, past smokers with pack-years of smoking 0–15, ≥15, current smokers), tumor stage, estrogen and progesterone receptor status, tamoxifen use, radiation and chemotherapy, and states of residence (Northern California, Southern California, Arizona, and Texas). Note: values in bold indicate that they are statistically or marginally different from the reference group.

**Table 4:** Joint associations of the summary scores of red meat, fruit, and vegetable intakes and seasons with C-reactive protein in different subgroups.

Joint Associations of Food Inflammatory Scores and Seasons with C-Reactive Protein in Different Subgroups										
		Beta (*p*-Value)	Beta (*p*-Value)	Beta (*p*-Value)	Beta (*p*-Value)		Beta (*p*-Value)	Beta (*p*-Value)	Beta (*p*-Value)	Beta (*p*-Value)
**Smoking Status**	**Past smokers with pack-years ≥15 (*n* = 448)**					**Never smokers and past smokers with pack-years <15 (*n* = 2439)**				
	Food score	Summer	Fall	Winter	Spring	Food score	Summer	Fall	Winter	Spring
	High (0 to 1)	Ref	0.009 (0.9)	−0.07 (0.6)	0.23 (0.15)	High (0 to 1)	Ref	0.03 (0.70)	**−0.16 (0.03)**	−0.04 (0.56)
		*n* = 93	*n* = 87	*n* = 88	*n* = 77		*n* = 488	*n* = 495	*n* = 446	*n* = 430
	Low (−1 to −2)	−0.15 (0.48)	**−0.42 (0.06)**	**−0.46 (0.08)**	0.02 (0.9)	Low (−1 to −2)	**−0.20 (0.05)**	−0.14 (0.16)	−0.09 (0.93)	**−0.33 (0.003)**
		*n* = 33	*n* = 27	*n* = 18	*n* = 25		*n* = 178	*n* = 165	*n* = 115	*n* = 122
**Having Cardio-metabolic**	**Yes (*n* = 689)**					**No (*n* = 2385)**				
**Condition**	Food score	Summer	Fall	Winter	Spring	Food score	Summer	Fall	Winter	Spring
	High (0 to 1)	Ref	−0.17(0.16)	**−0.21 (0.09)**	−0.11 (0.4)	High (0 to 1)	Ref	−0.04(0.16)	**−0.14 (0.06)**	−0.03 (0.70)
		*n* = 144	*n* = 147	*n* = 127	*n* = 128		*n* = 471	*n* = 484	*n* = 439	*n* = 429
	Low (−1 to −2)	−0.14 (0.48)	−0.23(0.21)	−0.20 (0.37)	**−0.52 (0.01)**	Low (−1 to −2)	**−0.24 (0.01)**	**−0.20 (0.06)**	−0.06 (0.63)	**−0.25 (0.03)**
		*n* = 37	*n* = 46	*n* = 28	*n* = 32		*n* = 182	*n* = 151	*n* = 110	*n* = 119
**Obesity Status**	**Obese (*n* = 801)**					**Normal and overweight (*n* = 2273)**				
	Food score	Summer	Fall	Winter	Spring	Food score	Summer	Fall	Winter	Spring
	High (0 to 1)	Ref	**−0.18 (0.08)**	−0.15 (0.17)	−0.15 (0.15)	High (0 to 1)	Ref	0.07 (0.41)	**−0.20 (0.01)**	−0.01 (0.91)
		*n* = 182	*n* = 182	*n* = 167	*n* = 165		*n* = 433	*n* = 449	*n* = 399	*n* = 392
	Low (−1 to −2)	**−0.35 (0.06)**	−0.26(0.16)	0.13 (0.59)	**−0.50 (0.03)**	Low (−1 to −2)	**−0.29 (0.007)**	**−0.26 (0.02)**	−0.14 (0.3)	**−0.39 (0.001)**
		*n* = 35	*n* = 32	*n* = 19	*n* = 19		*n* = 184	*n* = 165	*n* = 119	*n* = 132

Covariates in the linear regression model included age at diagnosis, race/ethnicity, education level, menopausal status at baseline, total calorie intake, physical activity, body mass index, cardio-metabolic condition, smoking status (never smokers, past smokers with pack-years of smoking 0–15, ≥15, current smokers), tumor stage, estrogen and progesterone receptor status, tamoxifen use, radiation and chemotherapy, and states of residence (Northern California, Southern California, Arizona, and Texas). Note: cardio-metabolic condition was not adjusted in the regression model when we conducted stratified analyses by cardio-metabolic condition; the sample size of each subgroup is presented underneath the beta (*p*-value). Values in bold indicate that they are statistically or marginally different from the reference group.

## Data Availability

Some of the data presented in this paper are available online at https://library.ucsd.edu/dc/collection/bb7885740w (accessed on 7 February 2021).

## Supplementary Materials

The following are available online at https://www.mdpi.com/2077-0383/10/4/636/s1. Table S1: Food intakes and other characteristics according to seasons, Table S2: Seasons of dietary 24-h recalls and blood draws, Table S3: Joint associations of food intakes and season of blood draw with serum C-reactive protein among participants who provided 24-h recalls and blood samples during the same seasons (*n* = 1819). and Table S4: Joint associations of inflammatory food scores and season of blood draw among participants who provided dietary 24 h recalls and blood samples during the same seasons (current smokers and past smokers with pack-years of smoking ≥15 were excluded).
