# Supplementary material for: Interrelationship of Seasons with Inflammation, Red Meat, Fruit, and Vegetable Intakes, Cardio-Metabolic Health, and Smoking Status among Breast Cancer Survivors
Source: J Clin Med. 2021 Feb 7;10(4):636. doi: 10.3390/jcm10040636 (PMC7915094; doi:10.3390/jcm10040636)
Supplement: Supplementary file 1 [file jcm-10-00636-s001.pdf]

Supplemental Tables

Supplemental Table S1. Food intakes and other characteristics according to seasons

Name of variables	Summer	Fall	Winter	Spring	P-value
Physical activity (MET/week)	662.5 (172.5 - 1320)	495.0 (157.5 - 1180.0)	600.0 (157.5 - 1180.0)	585.0 (180.0 - 1215.0)	0.15
Total calorie intake	1699 (1450 - 1997)	1677 (1431 - 1957)	1670 (1420 - 1951)	1693 (1435 - 1996)	0.07
Red meat intakes (g/day)	79.8 (30.9 - 170.2)	74.2 (29.3 - 185.8)	91.9 (37.2 - 210.5)	92.5 (37.2 - 187.4)	0.8
Fruit intakes (serving/day)	2.6 (1.5 - 3.8)	1.9 (1.1 - 3.2)	1.7 (0.9 - 2.8)	2.0 (1.1 - 3.1)	<0.001
Vegetable intakes (serving/day)	2.7 (1.8 - 3.8)	2.5 (1.7 - 3.7)	2.5 (1.6 - 3.5)	2.6 (1.7 - 3.6)	0.01
Whole grain	1.0 (0.50 - 1.50)	1.0 (0.5 - 1.5)	1.0 (0.5 - 1.5)	1.0 (0.5 - 1.5)	0.82
Refined grain	1.83 (1.18 - 3.80)	1.9 (1.3 - 2.7)	2.1 (1.4 - 2.7)	1.9 (1.3 - 2.7)	0.08
Dietary fiber	19.9 (15.6 - 25.3)	19.6 (15.3 - 24.3)	19.8 (15.7 - 25.3)	20.2 (15.8 - 25.9)	0.27
Total saturated fatty acids	17.1 (12.4 - 22.9)	16.5 (11.5 - 22.4)	16.5 (11.8 - 22.8)	16.5 (12.4 - 22.4)	0.65
Total monounsaturated fatty acids	20.2 (15.2 - 26.7)	19.2 (14.3 - 25.8)	19.7 (14.5 - 26.4)	20.1 (15.2 - 26.5)	0.14
N-3/N-6 fatty acid ratio	0.24 (0.18-0.30)	0.23 (0.18-0.29)	0.23 (0.18-0.30)	0.23 (0.19-0.29)	0.28
Trans fatty acids	2.6 (1.8 - 3.8)	2.6 (1.7 - 3.9)	2.8 (1.7 - 3.9)	2.7 (1.7 - 4.0)	0.63
Cholesterol	188.5 (134.0 - 261.0)	182.0 (123.0 - 260.0)	184.0 (119.0 - 256.0)	184.0 (131.0 - 259.0)	0.27
Alcohol	0.10 (0.0 - 6.1)	0.2 (0 - 6.9)	0.2 (0 - 7.3)	0.2 (0 - 6.9)	0.92
Zinc	9.0 (7.3 - 11.0)	8.8 (7.1 - 10.7)	8.6 (7.0 - 10.6)	8.9 (7.2 - 11.2)	0.04
Selenium	100.1 (82.0 - 120.9)	96.8 (80.2 - 117.6)	96.0 (78.7 - 115.4)	98.8 (82.3 - 118.9)	0.72
Thiamin	1.6 (1.3 - 1.9)	1.5 (1.3 - 1.8)	1.5 (1.3 - 1.8)	1.6 (1.3 - 1.9)	0.31
Riboflavin	1.7 (1.4 - 2.1)	1.7 (1.3 - 2.1)	1.7 (1.3 - 2.0)	1.7 (1.4 - 2.1)	0.01
Niacin	20.8 (17.0 - 25.4)	20.3 (16.6 - 24.3)	19.4 (16.0 - 23.7)	19.9 (16.4 - 24.3)	<0.0001
Folic acid	311.0 (241.0 - 405.0)	296.0 (223.0 - 382.0)	290.0 (221.0 - 385.0)	327.0 (253.0 - 422.0)	0.001
Potassium	2949.5 (2406.0 - 3465.0)	2831.0 (2295.0 - 3408.0)	2777.0 (2298.0 - 3355)	2914.0 (2385.0 - 3448.0)	0.0004
Magnesium	302.0 (250.0 - 366.0)	290.0 (241.0 - 357.0)	297.0 (244.0 - 360.0)	305.0 (251.0 - 371.0)	0.02
Iron	13.9 (11.3 - 17.5)	14.1 (11.3 - 17.1)	13.8 (11.1 - 17.1)	14.4 (11.6 - 17.8)	0.06

Vitamin A	1501.5 (898.0 - 4012.0)	1273 (795.0 - 2208.0)	1393.0 (836.0 - 2526.0)	1477.0 (881.0 - 2952.0)	<0.0001
Vitamin C	139.0 (92.0 - 197.0)	128.0 (87.0 - 179.0)	127.0 (82.0 - 186.0)	141.0 (95.0 204.0)	0.01
Vitamin B6	1.9 (1.6 - 2.3)	1.8 (1.5 - 2.3)	1.8 (1.4 - 2.2)	1.8 (1.5 - 2.4)	0.59
Vitamin B12	3.5 (2.4 - 4.9)	3.3 (2.2 - 4.9)	3.2 (2.1 - 4.6)	3.4 (2.3 - 5.0)	0.18
Vitamin D	3.9 (2.6 - 6.0)	4.0 (2.5 - 6.1)	4.1 (2.5 - 6.1)	4.0 (2.6 - 6.0)	0.69
Beta carotene	4081.0 (2289.1 - 7134.0)	4175.0 (2111.0 - 7353.0)	4349.0 (2112.0 - 7468.0)	4385.0 (2251.0 - 7869.0)	0.83
Alpha carotene	495.0 (134.1 - 1249.0)	533.1 (114.7 - 1435.8)	647.6 (166.2 - 1440.1)	673.2 (142.0 1609.3)	0.27
Alpha tocopherol	8.5 (6.7 - 11.5)	8.1 (6.3 - 10.4)	7.9 (6.2 - 10.4)	8.5 (6.7 - 10.7)	0.001
Caffeine	126.5 (43.5 - 249.2)	117.5 (30.8 - 239.6)	130 (39.9 - 254.0)	129.6 (41.0 - 257.6)	0.19

Supplemental Table S2. Seasons of dietary 24-hour recalls and blood draws

	**Season of dietary 24-hour recalls**	**Season of blood draws**	**Women who had blood draws and 24-hour recalls in the same season**
**Summer (N, %)**	873 (29.9%)	795 (27.2%)	514 (17.6%)
**Fall (N, %)**	657 (22.5%)	783 (26.8%)	425 (14.6%)
**Winter (N,%)**	643 (22.0%)	665 (22.8%)	427 (14.6%)
**Spring (N, %)**	739 (25.3%)	675 (23.2%)	453 (15.5%)

Supplemental Table S3. Joint associations of food intakes and season of blood draw with serum C-reactive protein among participants who provided 24-hour recalls and blood samples during the same seasons (n =1819).

		Beta (P-value) Summer	Beta (P-value) Winter	Beta (P-value) Fall and spring
**Red meat intakes**	> Quartile 1 (≥ 23 g/day)	Ref	**-0.15 (0.11)**	-0.01 (0.9)
	Quartile 1 (0 to <23 g/day)	-0.08 (0.5)	**-0.22 (0.14)**	**-0.29 (0.0009)**
**Fruit intakes**	Quartile (1-2)	Ref	**-0.25 (0.03)**	-0.09 (0.37)
	Quartile (3-4)	**-0.19 (0.09)**	**-0.20 (0.13)**	**-0.23 (0.03)**
**Vegetable intakes**	Quartile (1-2)	Ref	**-0.25 (0.03)**	-0.09 (0.37)
	Quartile (3- 4)	**-0.19 (0.09)**	**-0.20 (0.13)**	**-0.23 (0.03)**

Covariates in the linear regression model included age at diagnosis, race/ethnicity, education level, menopausal status at baseline, total calorie intake, physical activity, body mass index, cardio-metabolic condition, smoking status (never smokers, past smokers with pack-years of smoking 0-15, ≥15, current smokers), tumor stage, estrogen and progesterone receptor status, tamoxifen use, and states of residence (Northern California, Southern California, Arizona and Texas).

Supplemental Table S4. Joint associations of inflammatory food scores and season of blood draw among participants who provided dietary 24 hour recalls and blood samples during the same seasons (current smokers and past smokers with pack-years of smoking ≥15 were excluded)

Food score	Beta (P-value) Summer	Beta (P-value) Winter	Beta (P-value) Fall or Spring
High (0 to 1) (N= 1017)	Ref	**-0.16 (0.06)**	0.02(0.78)
Low (-1 to -2) (N=179)	**-0.17 (0.05)**	0.09(0.67)	**-0.34 (0.003)**

Covariates in the linear regression model included age at diagnosis, race/ethnicity, education level, menopausal status at baseline, total calorie intake, physical activity, body mass index, cardio-metabolic condition, smoking status (never smokers, past smokers with pack-years of smoking 0-15, ≥15, current smokers), tumor stage, estrogen and progesterone receptor status, tamoxifen use, and states of residence (Northern California, Southern California, Arizona and Texas).
